# Identification of multiple subclones in peripheral T-cell lymphoma, not otherwise specified with genomic aberrations

**DOI:** 10.1002/cam4.34

**Published:** 2012-09-26

**Authors:** Noriaki Yoshida, Akira Umino, Fang Liu, Kotaro Arita, Kennosuke Karube, Shinobu Tsuzuki, Koichi Ohshima, Masao Seto

**Affiliations:** 1 Division of Molecular Medicine, Aichi Cancer Center Research InstituteChikusa-ku, Kanokoden 1-1, Nagoya, 464-8681, Japan; 2 Department of Cancer Genetics, Nagoya University Graduate School of MedicineShowa-ku, Tsurumai 65, Nagoya, 466-8550, Japan; 3 Hematology and Oncology, Mie University Graduate School of MedicineEdobashi 2-174, Tsu, Mie, 514-8507, Japan; 4 Third Department of Internal Medicine, Graduate School of Medicine and Pharmaceutical Sciences, University of ToyamaSugitani 2630, Toyama, 930-0194, Japan; 5 Department of Pathology, School of Medicine, Kurume UniversityAsahimachi 67, Kurume, Fukuoka, 830-0011, Japan

**Keywords:** Multiple subclones, not otherwise specified, oligo-array comparative genomic hybridization, peripheral T-cell lymphoma

## Abstract

Peripheral T-cell lymphoma, not otherwise specified (PTCL, NOS) with genomic aberrations has been shown to resemble lymphoma-type adult T-cell leukemia/lymphoma (ATLL) in terms of its genomic aberration patterns, histopathology, and prognosis. We have shown recently that a majority of patients with acute-type ATLL have multiple subclones that were likely produced in lymph nodes. In this study, we analyzed whether PTCL, NOS with genomic aberrations also has multiple subclones as found in ATLL by means of high-resolution oligo-array comparative genomic hybridization (CGH). Thirteen cases of PTCL, NOS were available for 44K high-resolution array CGH analysis. The results showed that 11 (84.6%) of the 13 cases had a log2 ratio imbalance, suggesting that multiple subclones exist in PTCL, NOS with genomic aberrations. In order to analyze the association between multiple subclones and prognosis, we used previous bacterial-artificial chromosome (BAC) array analyses for 29 cases and found that the existence of multiple subclones was associated with a poor prognosis (*P* = 0.0279).

## Introduction

Peripheral T-cell lymphoma, not otherwise specified (PTCL, NOS) is one entity of the non-Hodgkin lymphomas referenced in the World Health Organization (WHO) classification [1]. It is a heterogeneous group of nodal and extra-nodal mature T-cell lymphomas that do not belong to any of the recognized entities in the T-cell lymphoma subtypes. Patients with PTCL, NOS have poor prognosis, although they do better than patients with adult T-cell leukemia/lymphoma (ATLL). It is reported that a high score for the international prognosis index (IPI) and a large number of transformed cells following histopathological analysis are related to poor prognosis [2, 3]. ATLL is a peripheral T-cell neoplasm caused by human T-cell leukemia virus type 1 (HTLV-1). Among its four clinical subtypes [4], acute and lymphoma types show extremely poor prognosis. Clinically, the IPI score is thought to have predictive value for patients with ATLL [5].

We have previously shown that approximately 70% of patients with acute-type ATLL have multiple subclones, which are derived from a common original clone [6]. The multiple subclones with accumulated different genomic aberrations were most likely produced in the lymph node as a result of clonal evolution. Our previous study using custom-made bacterial-artificial chromosome (BAC) array comparative genomic hybridization (CGH) revealed that PTCL, NOS with genomic aberrations resembles lymphoma-type ATLL not only in its genomic aberration patterns but also in terms of histopathology, cell surface marker, and prognosis. Thus, we demonstrated that a part of PTCL, NOS exhibits similar clinicopathological characteristics to ATLL [7]. In this study, we investigated in detail whether PTCL, NOS with genomic aberrations also has multiple subclones as in ATLL by performing high-resolution oligo-array CGH (Agilent Technologies, CA) using a 44000 probe set.

## Materials and Methods

### PTCL, NOS patients and samples

PTCL, NOS samples of lymph node with genomic aberrations detected by BAC array CGH in our previous study were used for this study [7]. Among the 29 cases with genomic aberrations, 13 were available for 44K high-resolution array CGH analysis. This study was performed under a protocol approved by the institutional review board of the Aichi Cancer Center. The clinical and pathological characteristics of patients are summarized in Table 1. Nuclear size was determined as comprising small, medium, large, anaplastic, or pleomorphic nuclei by two expert hematopathologists [7]. The 13 samples analyzed in this study have been proven to be monoclonal by Southern blot analysis as described in the previous report [7].

**Table 1:** Patients information and results of array CGH

PTCL no.^1^	Age (y.o.)	Sex	IPI	TCR rearrangement	Nuclear size^1^,^2^	Genomic aberrations	Genomic imbalance
27	64	Male	3 HI	R	2	Positive	Positive
29	76	Female	3HI	R	4	Positive	Positive
33	70	Male	4 HI	R	5	Positive	Positive
35	69	Female	2 LI	R	4	Positive	None
36	43	Female	N	R	5	Positive	Positive
39	48	Male	3 HI	R	4	Positive	Positive
43	57	Male	3 or 4	R	5	Positive	Positive
44	53	Male	3 LI	R	5	Positive	Positive
46	56	Male	2 HI	R	4	Positive	Positive
47	46	Male	1 L	R	5	Positive	Positive
48	79	Male	4 HI	R	5	Positive	Positive
50	80	Female	4 HI	R	2	Positive	None
51	NA	Female	2 LI	R	5	Positive	Positive

IPI, International Prognostic Index; PTCL, peripheral T-cell lymphoma; NA, not available; R, rearrangement; NE, not evaluable; y.o., years old.

1 Representations in previous Nakagawa et al. [7] paper were used.

2 Numbers with 1–5 indicating small, medium, large, anaplastic, and pleomorphic nuclei, respectively.

### Oligo-array CGH and CGH data analysis

We performed Agilent 44K Whole Human Genome CGH analysis on these 13 samples. Normal human male genomic DNA obtained from a pool of eight normal male peripheral blood mononuclear cells (PBMCs) was used in all experiments as a reference sample. Analysis of the log2 ratio was conducted in accordance with our previous report [6]. Briefly, raw data text files were transferred to the Genomic Workbench v5.0 software (Agilent Technologies) and aberration regions were analyzed. Among these aberrations, we obtained average log2 ratios of regions where aberrations continued over 400 probes and showed a linear average ratio of >0.1 or <−0.1. The log2 ratio imbalance was defined when a patient had a different average log2 ratio for gain or loss regions. Gain regions with a ratio >0.6 and loss regions with a ratio <−1.0 were excluded because they are likely regions with high copy number amplification or homozygous loss.

We also analyzed the existence of multiple subclones in PTCL using previous results evaluated by BAC aCGH [7]. As previously reported, we defined a region of gain or loss as three contiguous probes showing gain (log2 ratio = 0.2–1.0) or loss (log2 ratio = −1.0 to −0.2) in the BAC array CGH [7]. Among these aberrations, we obtained average log2 ratios from regions where aberrations continued over 10 probes.

### Statistical analysis

Differences in average log2 ratios and the relationship between pathological findings and the existence of subclones were evaluated using a Welch test and Fisher's exact test, respectively. The correlation between prognosis and the existence of multiple subclones was determined by overall survival (OS). OS was defined as the time (months) from diagnosis to death from any cause, and the estimate of OS distribution was calculated using the Kaplan and Meier method. A *P*-value <0.05 was considered statistically significant in these tests.

### Microarray data accession number

The microarray data obtained in this study have been submitted to ArrayExpress and the assigned accession number is E-MTAB-998.

## Results

### Existence of multiple subclones in PTCL, NOS

**Figure 1:**
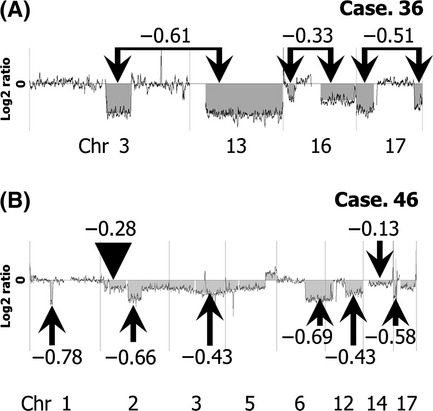
Genomic profiles of two cases with the log2 ratio imbalance evaluated by loss regions. (A) Array CGH result for Case 36. A typical log2 ratio imbalance was found in this case. We detected regions of loss on whole chromosomes 3, 13, 16, and 17. The log2 ratios of chromosomes 3 and 13 are −0.61, while those of chromosomes 16 and 17 are −0.33 and −0.51, respectively. (B) Case 46 showed a more complex log2 ratio imbalance for several chromosomes (arrows and arrowhead).

**Figure 2:**
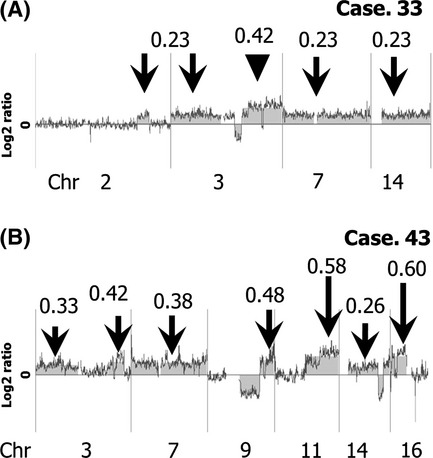
Genomic profiles of cases with the log2 ratio imbalance evaluated by gain regions. (A) Analysis of Case 33. Regions of gain involved chromosomes 2, 3, 7, and 14. The log2 ratio of 3q is 0.42 (arrowhead) and of other regions is 0.23 (arrows). (B) The log2 ratios of gain in Case 43 are more complex (arrows).

As expected, genomic aberrations were detected by 44K array CGH as previously detected by our custom-made BAC array CGH in all of the 13 PTCL, NOS samples analyzed (Fig. S1). Eleven (84.6%) of these samples showed log2 ratio imbalances (Table 1). Representative cases with log2 ratio imbalances are shown in Figures 1 and 2. In order to avoid complexity, the two cases in Figure 1 were evaluated by loss regions and the two cases in Figure 2 were evaluated by gain regions. The regions of loss were detected on chromosomes 3, 13, 16, and 17 in Case 36 (Fig. 1A). The log2 ratios of chromosomes 3 and 13 were −0.61, while those of chromosomes 16 and 17 were −0.51 and −0.33, respectively (Fig. 1A, arrows). The different log2 ratios suggest a log2 ratio imbalance in this case. Additionally, Figure 1B shows another case that had a more complex log2 ratio and imbalance.

In Case 33, there are regions of gain on chromosomes 2, 3, 7, and 14 (Fig. 2A). In this case, the log2 ratio average of 3q was 0.42 (arrowhead), while it was 0.23 (arrows) for other regions. Figure 2B shows representative genomic profiles of Case 43 with a more complex log2 ratio.

### Clonal evolution in PTCL, NOS

**Figure 3:**
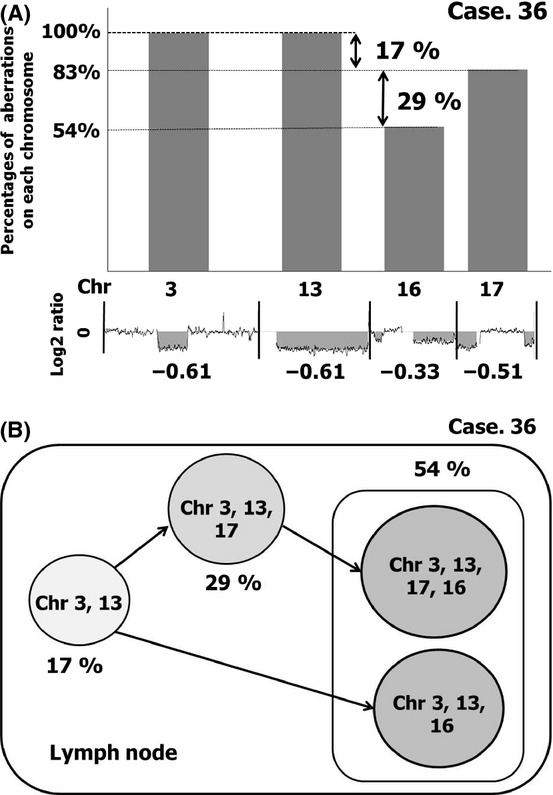
Possible schema of multiple subclones based on the different log2 ratios among chromosomes. (A) Case 36 is shown as monoclonal by T-cell receptor gene rearrangement. The average log2 ratio of aberrant regions reflected the ratio of tumors. In this case, all tumor cells are speculated to have regions of loss on chromosomes 3 and 13 based on the log2 ratio of the lowest level of −0.61. The log2 ratio of chromosome 17 is −0.51, which indicates that 83% (−0.51/−0.61) of tumor cells have a region of loss on chromosome 17. The log2 ratio of chromosome 16 is −0.33, which indicates that 54% (−0.33/−0.61) of tumor cells have a region of loss on chromosome 16. Thus, it is likely that 17% (100–83%) of tumor cells have losses only on chromosomes 3 and 13. Twenty-nine percent (83–54%) of tumor cells have losses on chromosomes 3, 13, and 17. The remaining 54% of tumor cells may comprise tumor cells with losses on chromosomes 3, 13, 17, and 16 or those with losses on chromosomes 3, 13, and 16. The ratio of these two subclones cannot be evaluated. (B) A model of clonal evolution in Case 36. According to the log2 ratio and imbalance in this case, the original tumor cell grows and accumulates genetic aberrations during clonal evolution as indicated by the arrows.

As previously demonstrated, the average log2 ratio of aberrant regions reflected the ratio of tumors [6]. In Case 36, all tumor cells have regions of loss on chromosomes 3 and 13. Fifty-four percent of tumor cells have a region of loss on chromosome 16, and 83% of tumor cells have loss on chromosome 17 (Fig. 3A). It is therefore speculated that clonal evolution took place as shown in Figure 3B.

### Histopathological features

Seven of the 13 patients had pleomorphic nuclei in tumor cells (Table 1). These patients tended to have a log2 ratio imbalance, although it did not reach a significant level (*P* = 0.192).

### Prognostic impact

**Figure 4:**
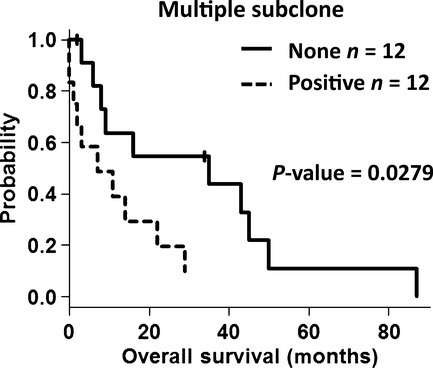
OS of patients with PTCL, NOS in relation to multiple subclones. The overall survival (OS) of PTCL, NOS patients with multiple subclones was inferior to that of patients without subclones.

As only 13 cases could be analyzed in this study, it was difficult to evaluate the relationship between prognosis and the existence of multiple subclones. We therefore used previous results obtained by BAC array CGH for analysis of this relationship in PTCL, NOS [7]. The clinical data of patients analyzed by BAC array CGH are summarized in Table S1 [7]. BAC array CGH consisted of 2304 probes for the genome, while 44K oligo-array CGH consisted of 44,000 probes. The results of both arrays were compared and found to be well correlated (Fig. S2) [8]. We evaluated the log2 ratio imbalance using the average log2 ratio of aberrations that continued over 10 probes for BAC array CGH. As a result, 12 of 29 PTCL, NOS samples had multiple subclones (Table S1). The OS of cases with multiple subclones was significantly inferior to that of cases without subclones in PTCL, NOS with genomic aberrations (Fig. 4) (*P* = 0.0279).

## Discussion

In this study, we found that the majority of patients with PTCL, NOS with genomic aberrations had multiple subclones in their lymph nodes. The majority of patients with ATLL also had multiple subclones in their lymph nodes [6]. The identification of multiple subclones in lymph nodes of PTCL, NOS as found in ATLL may further indicate the similarity between these diseases. It is therefore difficult to distinguish these two categories when HTLV-1 information is not available.

We detected the existence of subclones based on log2 ratio data. Both gain and loss regions are useful for the evaluation. Normal cells can make the absolute value of the log2 ratio lower, although they do not produce a log2 ratio imbalance. Thus, log2 ratio imbalances in cases that have been shown to be of monoclonal origin as identified by Southern blot analyses indicate the existence of multiple subclones. The fact that the log2 ratio average of aberrant regions reflects the ratio of tumors can help us follow the progression process of tumor cells [6]. For example, we could speculate that clonal evolutions in Case 36 took place as shown in Figure 3B on the basis of the log2 ratio imbalance and all of the subclones exist at diagnosis.

In this study, we found that samples with pleomorphic nuclei tended to have multiple subclones. It remains to be explored whether PTCL, NOS with a log2 ratio imbalance reflects the morphological features that resemble ATLL lymph node legions. The data analyzed by BAC array CGH were also useful for the detection of subclones in PTCL, NOS (Fig. S2). These results indicate the possibility that the existence of multiple subclones was associated with poor prognosis.

In the current WHO classification, PTCL, NOS represents a histologically and clinically heterogeneous group. Several studies have tried to categorize PTCL, NOS and suggested the presence of distinct subgroups [9–11]. It is known that both ATLL and PTCL, NOS with genomic aberrations express CC chemokine receptor (CCR) 4, while PTCL, NOS without genomic aberrations expresses CCR3. Anti-CCR4 antibody therapy for ATLL has now been applied to CCR4-positive PTCL, NOS [12]. Thus, our finding of the presence of multiple subclones in PTCL, NOS and ATLL suggests that these two disease categories partly share common clinicopathological features.

Recent whole-genome sequencing investigation of acute myeloid leukemia (AML) has revealed two possible scenarios [13]. One scenario indicates that the same original clone exists at diagnosis, while the other suggests that multiple subclones exist that were derived from the original clones with new mutations. At the relapse state after chemotherapies, one of these clones in tumor cells at diagnosis expanded. The same scenario may work in patients with PTCL, NOS with genomic aberrations and ATLL. It would therefore be important to analyze the relapsed samples of these diseases in detail. Thus, our finding of the presence of multiple subclones in PTCL, NOS and ATLL would be a great help in revealing the mechanism of lymphoma development, progression, and drug resistance in these diseases.

## Supporting Information

**Figure S1.** All genomic profiles of the 13 tested PTCL, NOS cases.

**Figure S2.** Comparison of 44K oligo-array CGH and BAC array CGH.

**Table S1.** Identification of subclones by results of BAC array CGH.
