# Supplementary material for: Identification of multiple subclones in peripheral T-cell lymphoma, not otherwise specified with genomic aberrations
Source: Cancer Med. 2012 Sep 26;1(3):289–94. doi: 10.1002/cam4.34 (PMC3544466; doi:10.1002/cam4.34)
Supplement: Supplementary file 1 [file cam40001-0289-SD1.pdf]

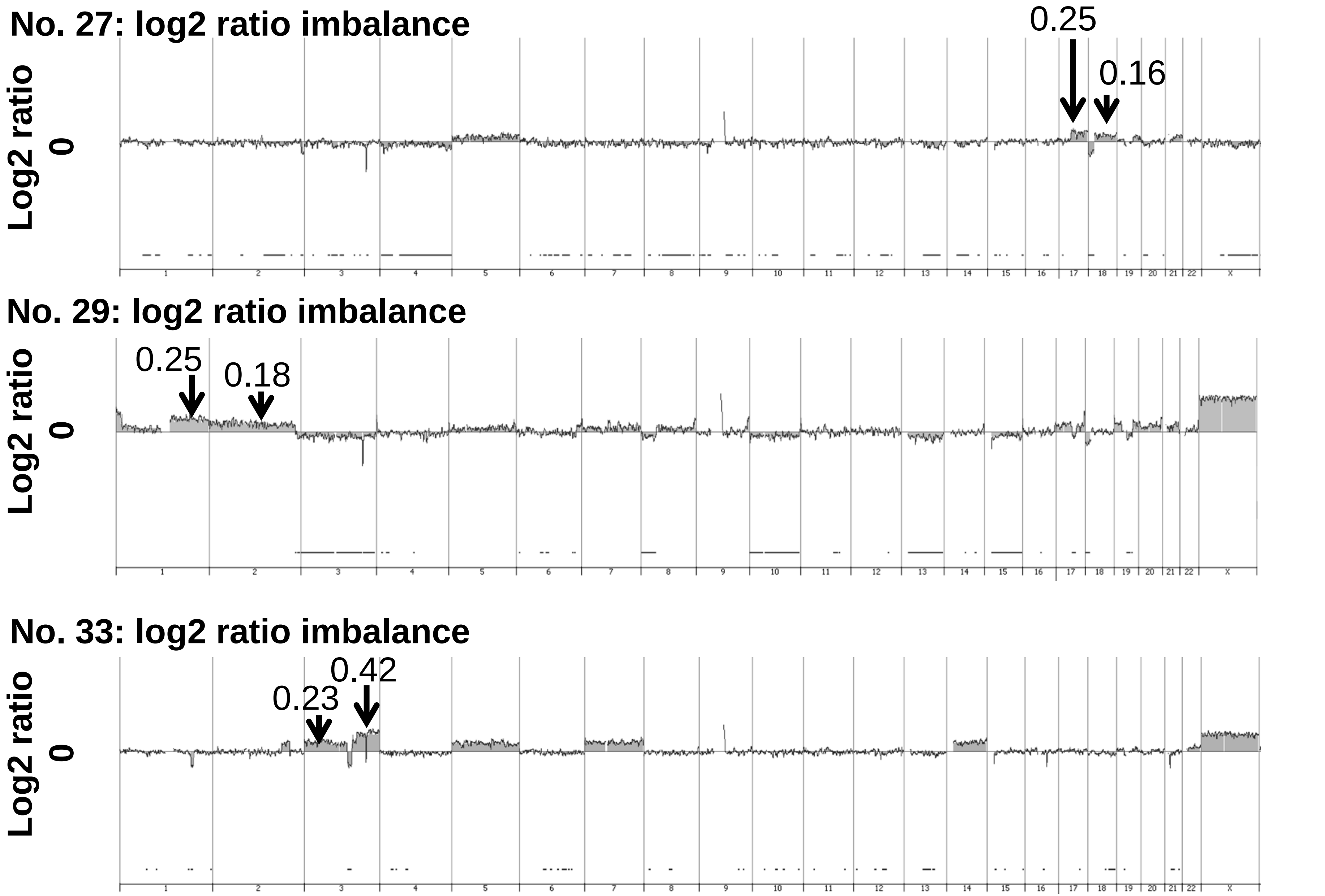

Supplemental Figure 1

All genomic profiles of the 13 tested PTCL, NOS cases.

No. 35: No log2 ratio imbalance

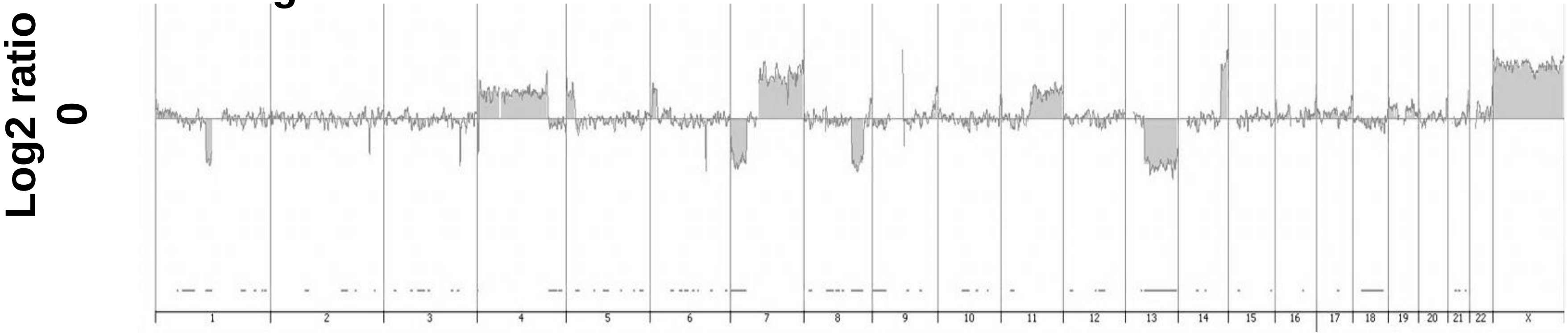

No. 36: log2 ratio imbalance

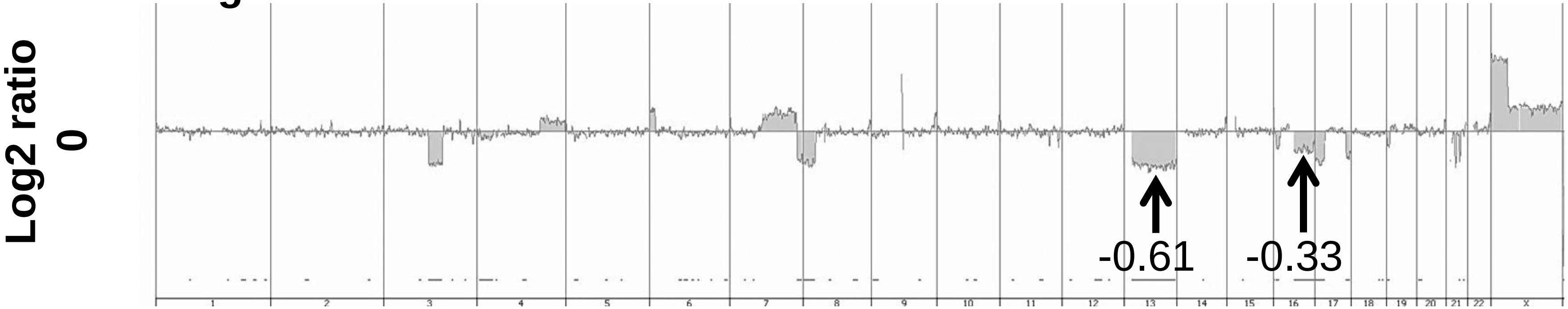

No. 39: log2 ratio imbalance

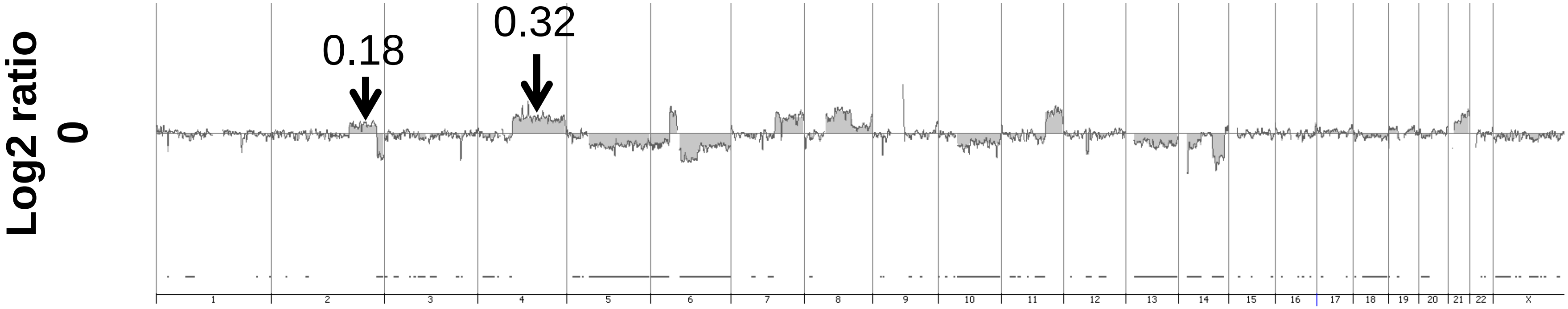

Supplemental Figure 1

All genomic profiles of the 13 tested PTCL, NOS cases.

No. 43: log2 ratio imbalance

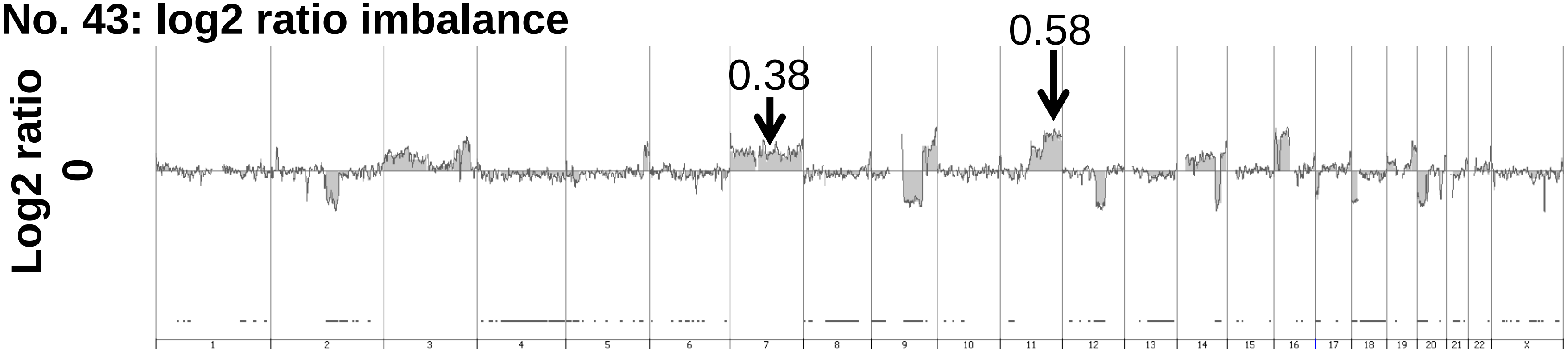

No. 44: log2 ratio imbalance

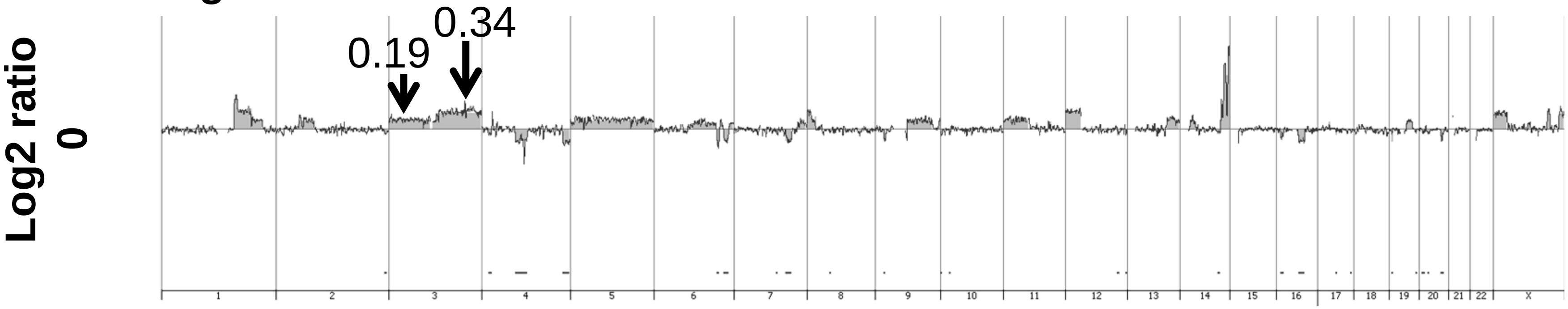

No. 46: log2 ratio imbalance

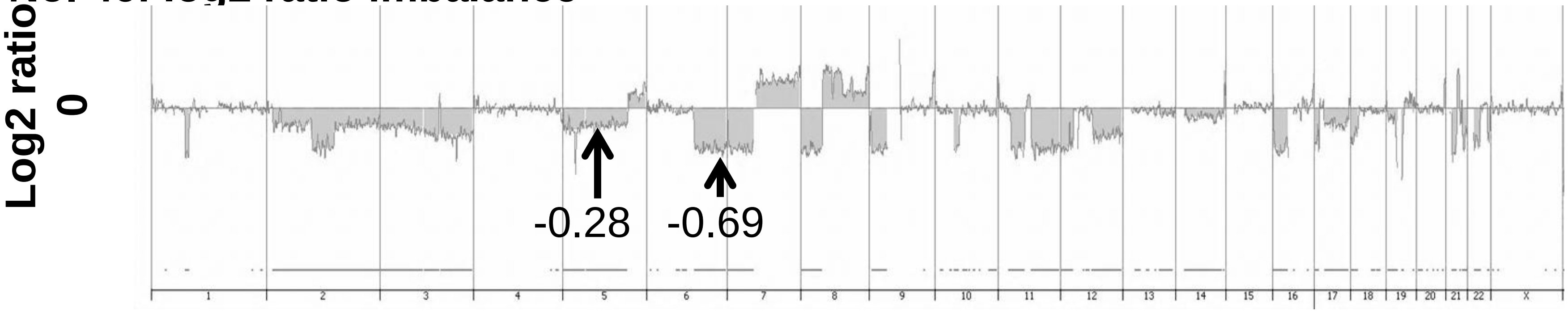

Supplemental Figure 1

All genomic profiles of the 13 tested PTCL, NOS cases.

No. 47: log2 ratio imbalance

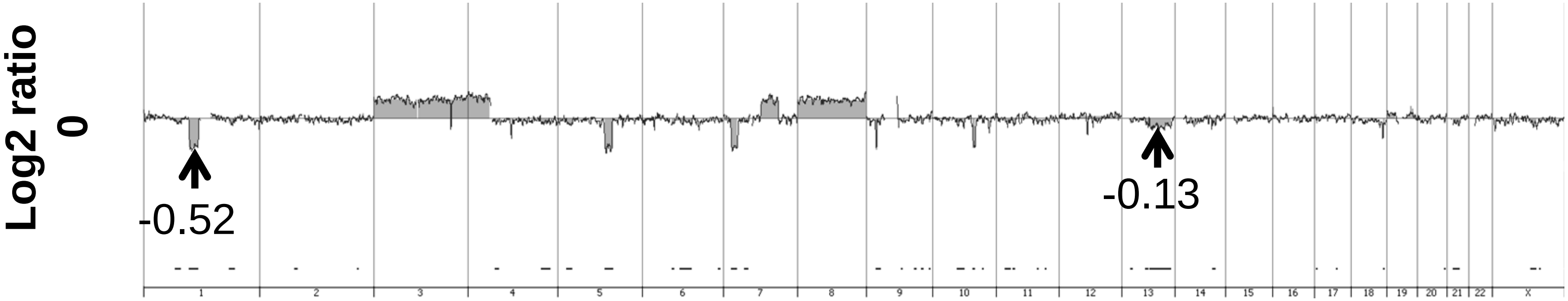

No. 48: log2 ratio imbalance

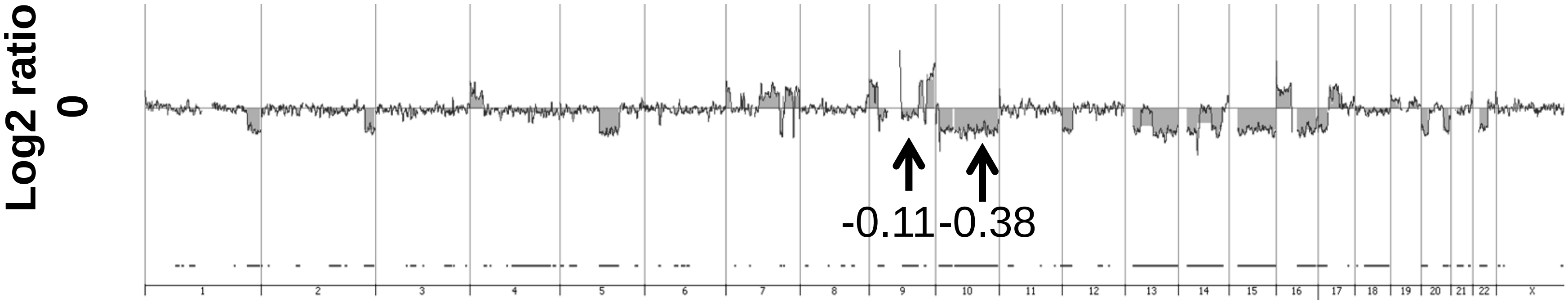

No. 50: No log2 ratio imbalance

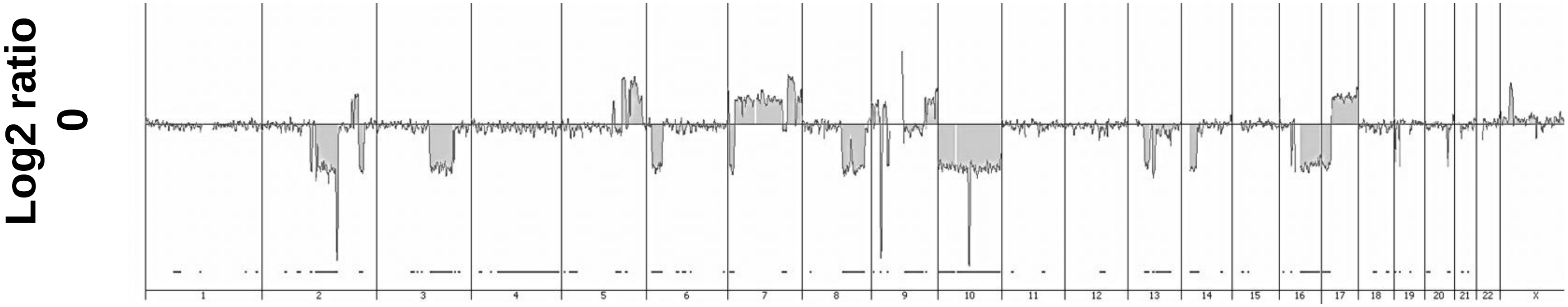

Supplemental Figure 1

All genomic profiles of the 13 tested PTCL, NOS cases.

No. 51: log2 ratio imbalance

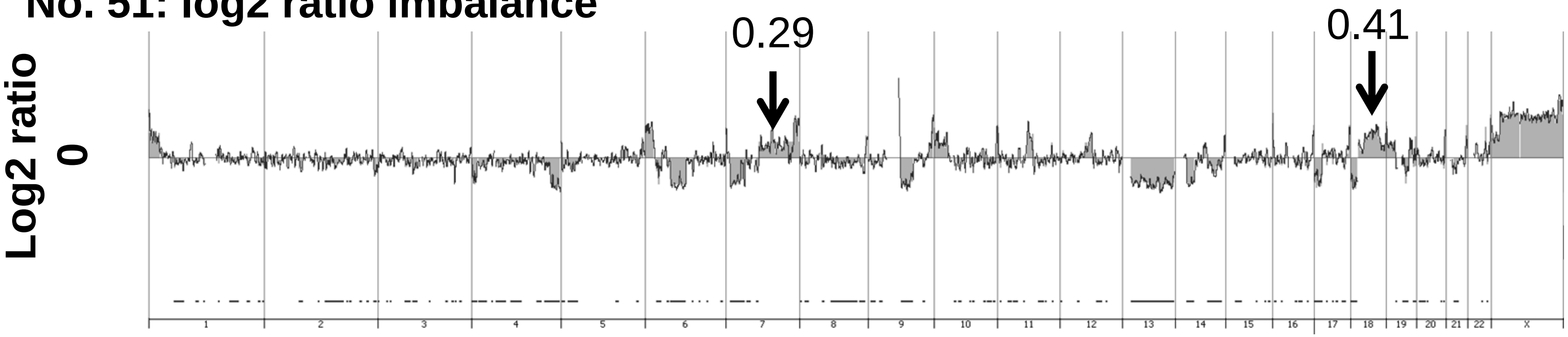

Supplemental Figure 1: All genomic profiles of the 13 tested PTCL, NOS cases. All cases have genomic aberrations. Eleven of the 13 (84.6%) cases have log2 ratio imbalances, suggesting the existence of multiple clones.

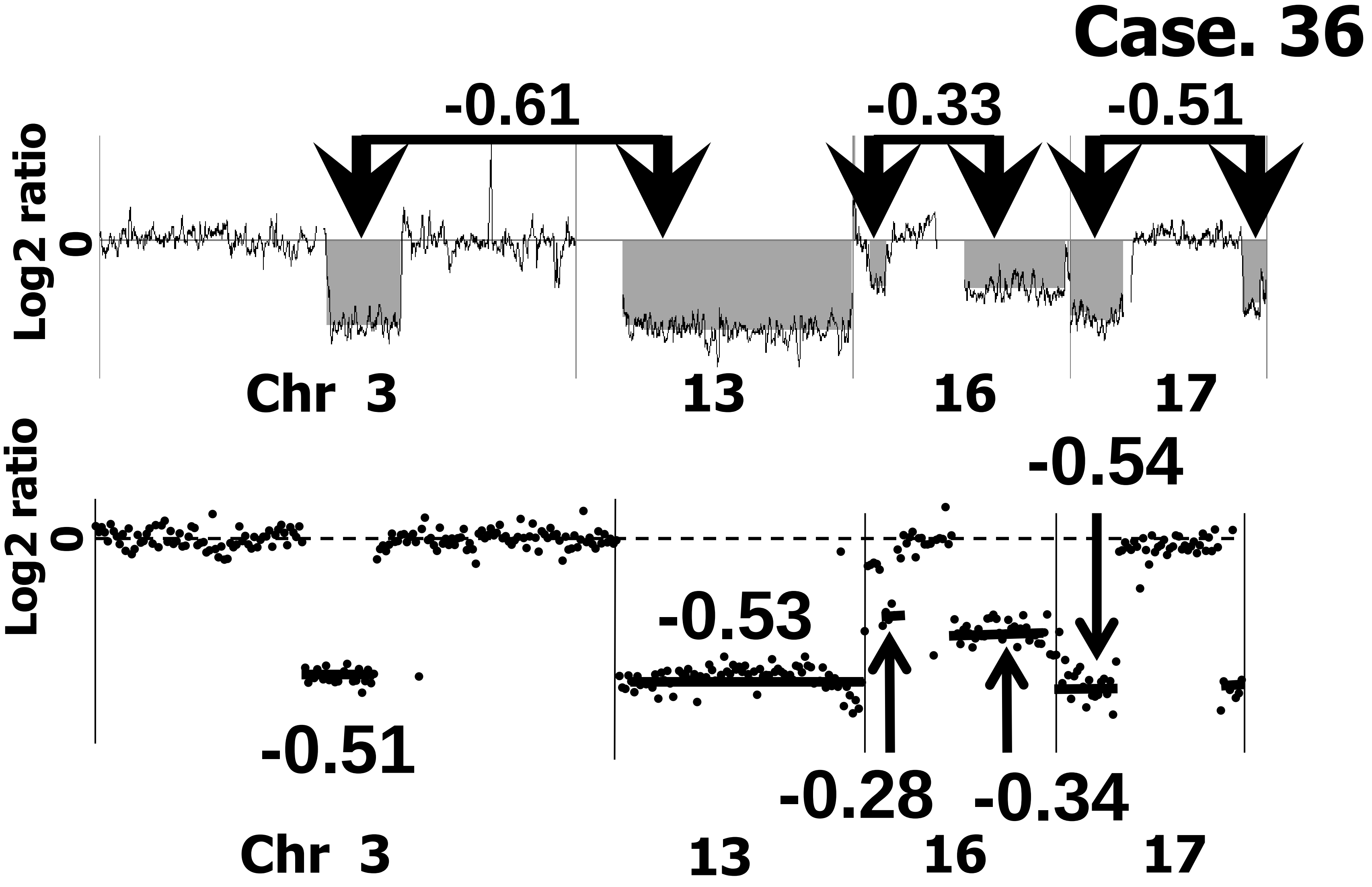

Supplemental Figure 2: Comparison of 44K oligo-array CGH and BAC array CGH at a same patient sample. Case 36 was analyzed by 44K oligo-array CGH (upper) and BAC array CGH (lower). The results of both arrays were well correlated. The log2 ratio imbalance was also detected in BAC array analyses.

Table S1 Identification of subclones by results of BAC array CGH

PTCL-No. *	observation period (months.) *	event *	genomic aberrations	existence of subclones
23	35	D	+	-
24	16	D	+	-
25	NA	NA	+	-
26	34	A	+	-
27	9	D	+	-
28	43	D	+	-
29	NA	NA	+	-
30	6	D	+	-
31	3	D	+	-
32	0	D	+	+
33	3	D	+	+
34	29	D	+	+
35	NA	NA	+	-
36	7	D	+	+
37	NA	NA	+	-
38	45	D	+	-
39	11	D	+	+
40	2	A	+	-
41	87	D	+	-
42	29	A	+	+
43	0	D	+	+
44	NA	NA	+	-
45	50	D	+	-
46	1	D	+	+
47	8	D	+	-
48	14	D	+	+
49	2	D	+	+
50	22	D	+	+
51	3	A	+	+

* Representations in previous Nakagawa et al. paper were used.

Abbreviations: NA; not available, A; alive D; dead, +; presence, −; absence
