# Supplementary figures and images for: Identification of multiple subclones in peripheral T-cell lymphoma, not otherwise specified with genomic aberrations
Source: Cancer Med. 2012 Sep 26;1(3):289–94. doi: 10.1002/cam4.34 (PMC3544466; doi:10.1002/cam4.34)

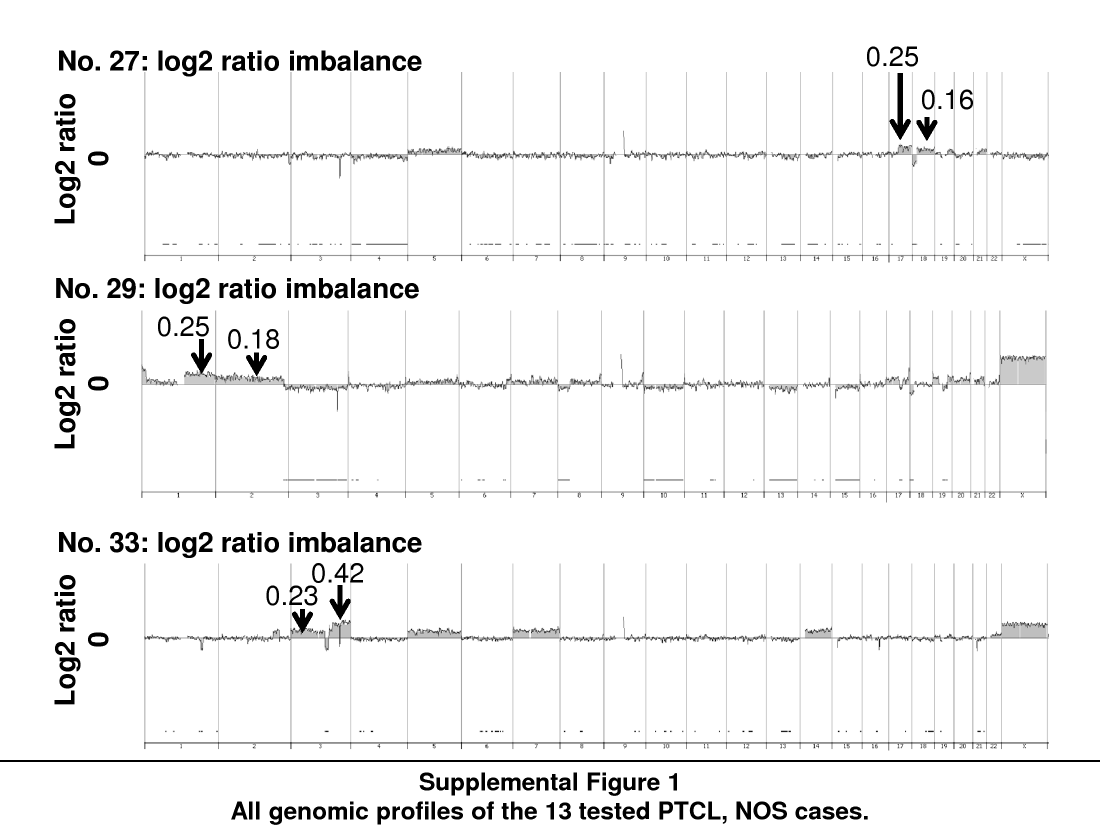

Supplement: Supplementary file 2 [file cam40001-0289-SD4.png]
